# Composite Nafion Membranes with CaTiO_3−δ_ Additive for Possible Applications in Electrochemical Devices

**DOI:** 10.3390/membranes9110143

**Published:** 2019-10-31

**Authors:** Lucia Mazzapioda, Maria Assunta Navarra, Francesco Trequattrini, Annalisa Paolone, Khalid Elamin, Anna Martinelli, Oriele Palumbo

**Affiliations:** 1 Department of Chemistry, Sapienza University of Rome, Piazzale Aldo Moro 5, 00185 Rome, Italy; lucia.mazzapioda@uniroma1.it (L.M.); mariassunta.navarra@uniroma1.it (M.A.N.); 2 Department of Physics, Sapienza University of Rome, Piazzale Aldo Moro 5, 00185 Rome, Italy; francesco.trequattrini@roma1.infn.it; 3 Consiglio Nazionale delle Ricerche, Istituto dei Sistemi Complessi, U.O.S. La Sapienza, Piazzale A. Moro 5, 00185 Roma, Italy; annalisa.paolone@roma1.infn.it; 4 Department of Chemistry and Chemical Engineering, Chalmers University of Technology, 41296 Gothenburg, Sweden; khalid.elamin@chalmers.se (K.E.);

**Keywords:** Nafion, CaTiO_3-δ_, inorganic filler, composite electrolyte

## Abstract

A composite membrane based on a Nafion polymer matrix incorporating a non-stoichiometric calcium titanium oxide (CaTiO_3−δ_) additive was synthesized and characterized by means of thermal analysis, dynamic mechanical analysis, and broadband dielectric spectroscopy at different filler contents; namely two concentrations of 5 and 10 wt.% of the CaTiO_3−δ_ additive, with respect to the dry Nafion content, were considered. The membrane with the lower amount of additive displayed the highest water affinity and the highest conductivity, indicating that a too-high dose of additive can be detrimental for these particular properties. The mechanical properties of the composite membranes are similar to those of the plain Nafion membrane and are even slightly improved by the filler addition. These findings indicate that perovskite oxides can be useful as a water-retention and reinforcing additive in low-humidity proton-exchange membranes.

## 1. Introduction

Nafion is the archetypical membrane for use in the proton exchange membrane fuel cell (PEMFC), a clean technology suitable for both transport and stationary applications [1,2]. However, to be more efficient electrical power generators, fuel cells should operate at low relative humidity (RH) conditions and at temperatures higher than 80 °C. Indeed, at these conditions, the kinetics of the electrode reactions, the tolerance to fuel contaminants, such as carbon monoxide, and the ions’ transport properties are all improved. Furthermore, an increased operating temperature would mitigate the problems related to thermal and water management [3].

Unfortunately, in PEMFCs operating at temperatures above 80 °C, Nafion experiences a severe decrease in proton conductivity due to water evaporation, which is also reflected in an increase of the electrolyte’s ohmic resistance [4].

One strategy to develop PMFC electrolytes suitable for high-temperature operation is to modify the polymer matrix with inorganic additives that are able to improve the water retention capacity of Nafion membranes. These additives, which can be metal oxide nanoparticles, such as SiO_2_, TiO_2_, and ZrO_2_, or functionalized inorganic materials, such as sulfated metal oxide, reinforce the hydration and proton conductivity of the membranes, thanks to their acidic and hygroscopic properties, allowing them to work at the desired conditions described above [5,6,7,8]. In particular, the incorporation of hygroscopic particles in the host polymer can increase the accessible surface in Nafion membranes, facilitating water trapping and creating an additional way for the transfer of protons through the membrane, hence improving the overall fuel cell performance at high temperatures.

Titanium-based oxides are very attractive materials, thanks to their good features in terms of cost, stability, and high natural abundance, triggering the R&D toward the improvement of their chemical–physical properties by the study of their chemistry and surface geometry, making them useful to several research fields [9]. In particular, oxides with a perovskite structure are materials that have found the potential for a wide range of applications, such as sensors, optical devices, and solid-oxide-fuel-cells electrodes and electrolytes [10]. This could be explained by the flexibility of the perovskite design (ABO_3_), which allows for the accommodation of several doping agents, such as metal transition elements at both A- and/or B-sites in the lattice. This leads to changes of the electrical and optical properties of the oxide, introducing new electronic levels in the energy gap and providing oxygen vacancies in the lattice of the perovskite [11].

It is expected that the main features of perovskites are controlled by the size and nature of both A and B cations in the ABO_3_ structure and that the occupation of the B-site by several ions with different acid/base properties affects the stability of the perovskite. Furthermore, the presence of the oxygen vacancies in the structure can play a key role as active sites for the oxygen adsorption and the dissociative absorption of water, for which the protons conductivity is favored. The last aspect could be used to improve the hydrophilicity of the oxide by the protonation of the lattice oxygen ions. This phenomenon seems to be predominant in perovskite-type oxides due to the low formation enthalpies of oxygen ions as a consequence of low bond strengths and strong relaxation effects [12,13,14].

Consequently, with the aim to combine all the features of the abovementioned materials, a non-stoichiometric perovskite titanium oxide, calcium titanate (CaTiO_3−δ_), is here proposed as a water-retention and reinforcing additive in low-humidity proton-exchange membranes. The scope of the present study is to investigate the impact of the additive on thermomechanical and proton-conduction properties of membranes by differential scanning calorimetry (DSC), dynamic mechanical analysis (DMA), and broadband dielectric spectroscopy (BDS) studies, in order to evaluate the interplay of the transition/relaxation phenomena in Nafion membranes at high temperatures and under humidified conditions.

## 2. Materials and Methods 

The CaTiO_3−δ_ additive was prepared by a template-driven procedure recently proposed by our group [14], using Pluronic F127 to control the structure of the particles and to promote the formation of oxygen vacancies. More specifically, titanium isopropoxide and calcium dichloride dehydrated were used as precursors. Pluronic F127 was dissolved in ethanol (molar ratio1:4), under vigorous stirring, for 20 min, at 60 °C. Subsequently, titanium isopropoxide was added into the above solution, to obtain a titanium-oxide-based sol. At the same time, calcium dichloride was dissolved in deionized water, and, after 15 min, it was added into the mixture. A 3M NaOH solution was dripped to facilitate the complete dissolution of TiO_2_ and its conversion to CaTiO_3_. The solution was transferred in an autoclave and treated at 180 °C, for 24 h, followed by natural cooling to room temperature. After that, the solid product was centrifuged and washed several times with bi-distilled water. Finally, the perovskite was calcinated for 3 h, at 550 °C (heating rate 3 °C/min), to remove the occluded template. During this step, the polymer was oxidized, creating a reductive environmental near the oxide surface and facilitating the formation of oxygen defects. All the reagents for this synthesis were Sigma-Aldrich products (Sigma-Aldrich, St. Louis, MO, USA).

The sample particles obtained have well-defined prismatic, quasi-cubic morphology, as well as the presence of holes/imperfections on the perovskite surface, which can be observed. Based on Brunauer–Emmett–Teller (BET) analysis, the specific surface area was found to be 6.6 ± 0.5 m^2^·g^−1^.

A solvent-casting procedure was used to prepare both doped and undoped Nafion membranes, according to an established procedure [15,16,17]. Solvents of a commercial Nafion 5 wt.% dispersion (E.W. 1100, Ion Power Inc., München, Germany) were gradually replaced with N,N-dimethylacetamide (>99.5%, Sigma-Aldrich, St. Louis, MO, USA), at 80 °C. For the composite membranes, two filler concentrations of 5 and 10 wt.% of the CaTiO_3−δ_ additive, with respect to the dry Nafion content, were chosen and added to the final Nafion solution. The mixture obtained was casted on a Petri dish and dried at 80 °C. In order to improve the thermal stability and robustness of the membranes, dry membranes were extracted and hot-pressed at 50 atm, 175 °C, for 15 min. The membranes were activated and purified in boiling 3 wt.% hydrogen peroxide (H_2_O_2_, 34.5–36.5%, Sigma-Aldrich, St. Louis, MO, USA), H_2_SO_4_ (0.5 M) and distilled water. Composite membranes were compared to plain Nafion systems prepared with the same procedure. All samples were stored in bi-distilled water. Membranes containing 5 and 10 wt.% of the inorganic filler are labeled in the text as M5 and M10, respectively, while the undoped membrane is referred to as N.

The mechanical properties of the membranes were measured by means of a DMA 8000 (Perkin Elmer Waltham, MA, USA) in the so-called “tension configuration”, on small membrane pieces that were 4–6 mm wide, 10–12mm long, and 0.10–0.15mm thick [18,19,20,21]. The samples were cut from the various membranes dried in an oven, at 80 °C (dry samples), or immersed in bi-distilled water, at room temperature (wet samples). In this latter case, to prevent the release of water, the samples were very quickly mounted into the DMA apparatus and measured. The storage modulus, *M*, and the elastic energy dissipation, tanδ, were measured at 1 and 10 Hz, as a function of temperature between 20 and 190 °C, with a scan rate of 4 °C/min.

DSC experiments were carried out using a DSC821 instrument (Mettler-Toledo, Zaventem, Belgium), under nitrogen (N_2_) flux (60 mL/min), in a temperature range between 30 and 150 °C, at a scan rate of 20 °C/min. Before DSC measurements, membrane samples were equilibrated at 100% relative humidity (RH) for two weeks. By using the STARe software, the determination of both the T_onset_ and the enthalpy values associated with the thermal transition were evaluated. In particular, the T_onset_ was defined as the intersection of the tangent of the peak with the extrapolated baseline, whereas the peak area was proportional to the enthalpy of the thermal event.

Thermal gravimetric (TG) analysis was performed on dry samples, with a TGA/SDTA851 (Mettler-Toledo, Zaventem, Belgium), under air (80 mL/min), in a temperature range between 25 and 550 °C. Prior to measurements, the samples were dried at 80 °C, under vacuum, overnight.

Dielectric measurements were performed using a NovocontrolGmBH broadband dielectric spectrometer (Montabaur, Germany), equipped with a QuatroCryosystem temperature-control unit. The membranes were placed between two carbon electrodes and then between two gold-plated electrodes (diameter of 10 mm), under humidity condition (i.e., we kept a water reservoir under the bottom electrode. The spectra were measured in the frequency range from 10^−1^ to 10^7^Hzand at different temperatures, with the following temperature sequence: 20 °C → 80 °C → 110 °C → 80 °C → 20 °C.

## 3. Results and Discussion

### 3.1. Differential Scanning Calorimetry and Thermal Gravimetric Analysis

**Figure 1:**
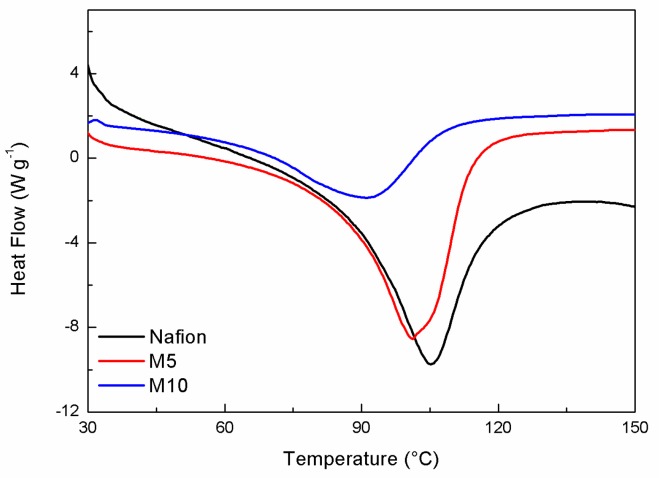
DSC response of the hydrated membrane samples.

The DSC curves of all membranes are displayed in Figure 1. In the investigated temperature range, a main peak is observed around 100 °C, assigned to an order–disorder transition of the ionic clusters in Nafion [22]. The enthalpy value, calculated by integrating the DSC peak, associated to this endothermic phenomenon, was evaluated and is reported in Table 1. In accordance to the literature [22], only water associated with Nafion hydrophilic groups contributed to this thermal transition. In particular, the enthalpy value increased with an increasing degree of hydration of the polymer, leading to a major organization of ionic clusters and more cohesive interactions. At the same time, the change in temperature of the transition peak is attributed to a plasticizing effect of water, for which a shift toward lower temperatures may correspond to higher hydration levels. However, in our case, the change in the enthalpy values is much more significant than that in *T*_onset_.

Among all samples, the composite membrane M5 (containing 5 wt.% of the CaTiO_3−δ_ additive) shows a higher ΔH value than both M10 (10 wt.% of CaTiO_3−δ_) and, to a lower extent, N (plain Nafion) samples. The addition of CaTiO_3−δ_ particles caused an increase in the water content, even though the M10 sample, having the highest concentration of additive, displayed the lowest water affinity, possibly due to phase segregation and a non-optimized distribution of the inorganic additive [23,24], which can prevent the motions of the segments among the fluorocarbon backbone to restrict the water release.

**Figure 2:**
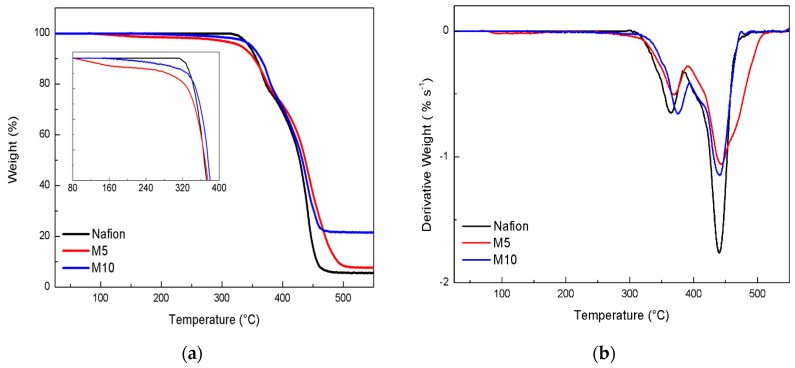
(**a**)TG and DTG curves (**b**) recorded for the dry membranes.

The TGA curves obtained for the three membranes are shown in Figure 2 (panel a). The thermal decomposition of the Nafion membrane occurs in three main steps. The first decomposition is associated with desulfonation of the side-chain of the polymer; the second and the third transitions, occurring in the range 350–450 °C, are related to side-chains and perfluorinated backbone decompositions [25].

The TGA profiles show that all the membranes are thermally stable up to 300 °C, even though, compared to plain Nafion, the two composite samples exhibit slightly higher decomposition temperatures, as better shown by the derivative curves (see DTG curves in Figure 2b). In particular, the last thermal process looks broader and shifted to higher temperatures for the M5 sample, suggesting a stabilizing interaction between the filler and the Nafion matrix. Moreover, as shown in the inset of TGA profiles (see Figure 2a), the composite membranes exhibit a smooth mass loss in a lower temperature range (below 300 °C), most likely due to traces of surface water still present in the membrane after the drying treatment carried out at 80 °C prior to the measurement. This can be explained in terms of extra hygroscopicity induced by the inorganic fillers. The presence of surface water looks to be more evident in M5.This could be explained by considering an optimal concentration/dispersion of the perovskite additive, able to hold water during the drying step and to release it gradually at higher temperature. The greater slope observed in M5, as compared to M10 (see inset of Figure 2a), suggests a higher content of surface water. Considering the final weights after TGA analysis, it is clear that the M10 membrane leaves a higher amount of residual weight, in respect to the other samples. This evidence could be interpreted by assuming a strong interaction between the Nafion polymer and the perovskite particles, for which the removal of the decomposed Nafion products at a high temperature is more difficult.

### 3.2. Dynamical Mechanical Analysis

**Figure 3:**
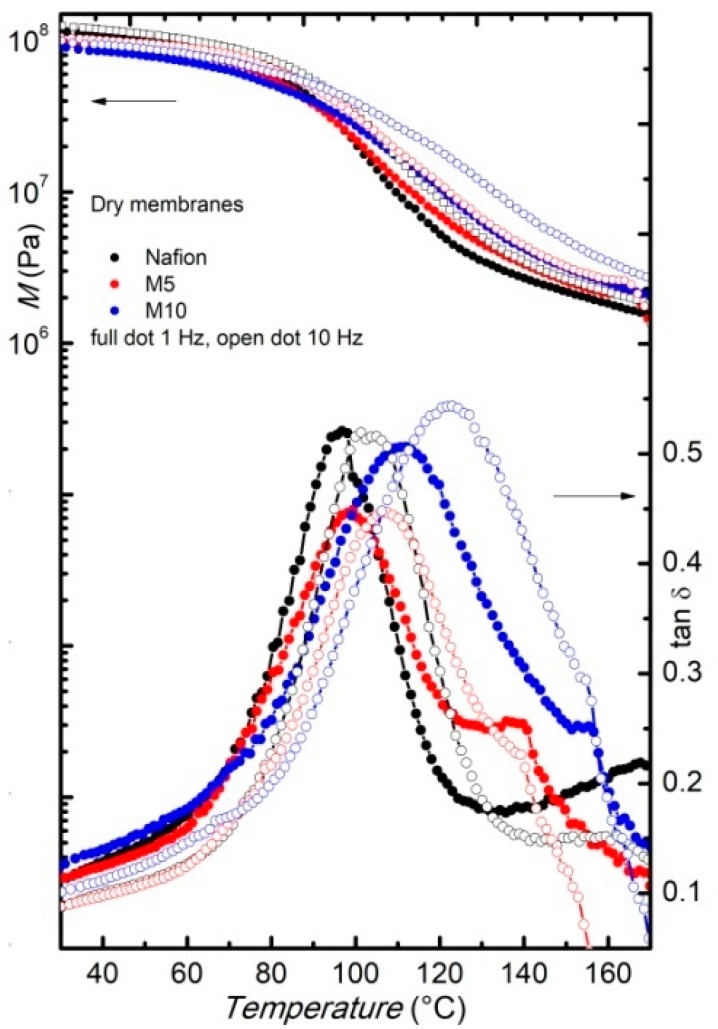
Modulus and elastic energy dissipation of pure and dry Nafion (black) and composite membranes M5 (red) and M10 (blue), measured on heating, at two frequencies, i.e., *f* = 1 Hz (full dots) and *f* = 10 Hz (open dots).

Figure 3 reports the storage modulus and the elastic energy dissipation of the dry membranes measured at two fixed frequencies (1 and 10 Hz), during heating between room temperature and180 °C, with arate of 4 °C/min. In agreement with DSC measurements, the Nafion membrane shows a relaxation around 110 °C, indicated by the occurrence of an intense peak in tanδ and a two-order magnitude drop in the modulus. This relaxation, usually indicated as α-relaxation, was already largely reported [18,19] and corresponds to the glass transition of the hydrophilic domains (polar regions) of Nafion [26].

In the composite membraneM5, the α-relaxation is slightly shifted to higher temperatures compared to the pure Nafion sample, and this shift is even more clear when increasing the amount of filler, since, for the M10 samples, the relaxation is observed at around 130 °C. At room temperature, the modulus values of the three samples are close, while at higher temperature, the composite membranes present slightly higher modulus values, thus confirming the reinforcing action of the filler. In particular, above 90 °C the modulus of the M10 membrane is the highest. Indeed, at room temperature, the modulus values could be affected by some undesired water contamination occurring during sample loading, while on heating above 100 °C, these effects should be suppressed. An increase of the modulus in a Nafion membrane, with an addition of a filler like SnO_2_ nanoparticles [27] and sulphated SnO_2_ ceramic nano-powders [19], was already observed.

**Figure 4:**
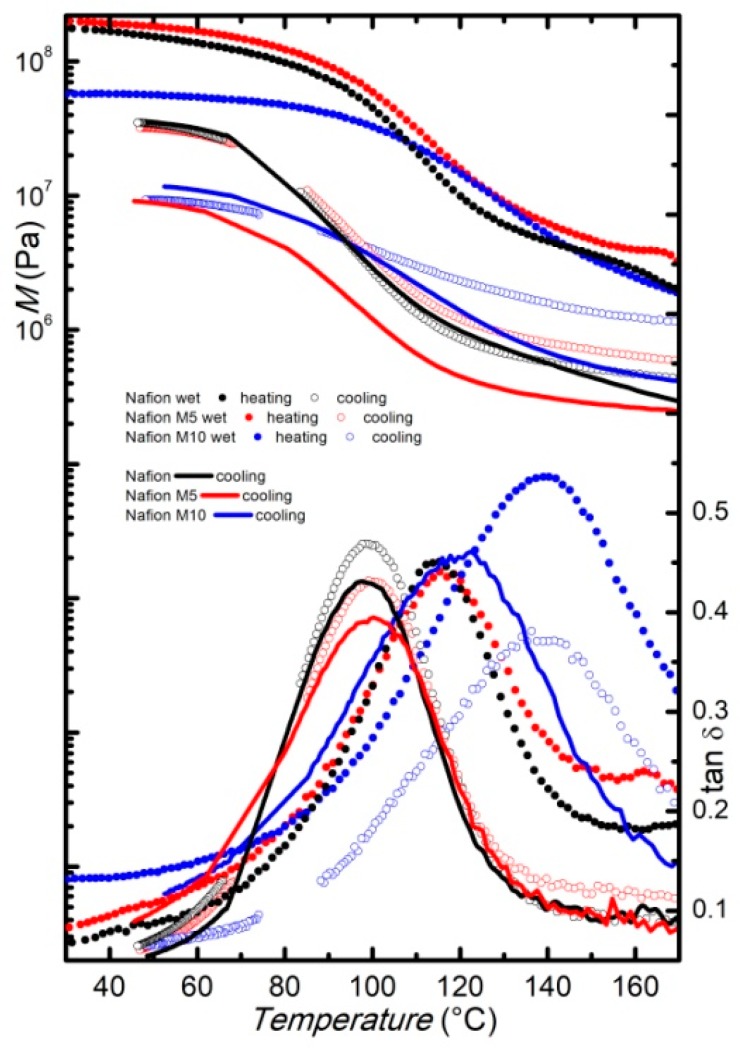
Modulus and elastic energy dissipation of pure Nafion (black) and composite membranes M5 (red) and M10 (blue) in the wet state, measured at *f* = 1 Hz, on heating (full dots) and subsequent cooling (open dots). Dry membranes measured at *f* = 1 Hz, on cooling (lines), are also reported for comparison.

The modulus and the elastic-energy dissipation of the wet membranes measured at a fixed frequency of 1 Hz during heating between room temperature and180 °C, with a rate of 4 °C/min, are reported in Figure 4. The α-relaxation is shifted to slightly higher temperatures compared to the dry membranes, and the highest temperature for the maximum of the energy dissipation is observed for the wet M10 membranes, confirming that the faded filler increases the relaxation temperature. On cooling (Figure 4), the peak associated to the α-relaxation presents its maximum at about 90 °C for the pure Nafion and the M5 membranes. In both membranes the α-relaxation displays a clear thermal hysteresis between heating and subsequent cooling, as already reported for similar systems [18,19].

Contrarily, the peak displayed by the M10 membrane does not present a significant temperature shift when measured on cooling. For comparison, the curves (both storage modulus and tanδ) measured on cooling for the dry samples are also reported in Figure 4. The curves measured in the cooling run for the Nafion and the M5 membranes, starting from their wet state, are close to the curves measured on cooling for the corresponding dry samples. Conversely, the tanδ measured on cooling for the M10 membranes, starting from the wet state, displays the α-relaxation at the same temperature at which it is detected in the heating run, and well above the temperature at which it appears in the spectrum measured on cooling, starting from the dry state. Indeed, it seems that, for this latter sample, the α-relaxation shows a very small thermal hysteresis between heating and cooling, regardless of the water content, maybe due to some interactions between the higher amount of filler and the Nafion matrix. Moreover, with a higher amount of filler, the presence of water (i.e., at wet conditions) seems to increase, more remarkably, the temperature at which this relaxation occurs.

The modulus values of the wet Nafion and M5 samples measured at room temperature before heating are close to that displayed by the corresponding membranes in the dry state, while the modulus displayed by the wet M10 membrane is slightly lower than the one measured on the dry M10 membrane, suggesting that, in this latter case, water acts more remarkably as a plasticizer that decreases the stiffness of the membranes. However, when comparing the modulus value in the cooling run, where most of the water may have evaporated, the curves are close for the same kind of samples, independent of the initial state (wet or dry). This confirms that the thermal treatment completely eliminates the hydration history of the samples. A small difference can be noticed only for the M5 membranes, where the modulus measured in the cooling run, starting from the wet state, is slightly higher than those obtained when cooling the dry sample.

### 3.3. Dielectric Spectroscopy Studies

**Figure 5:**
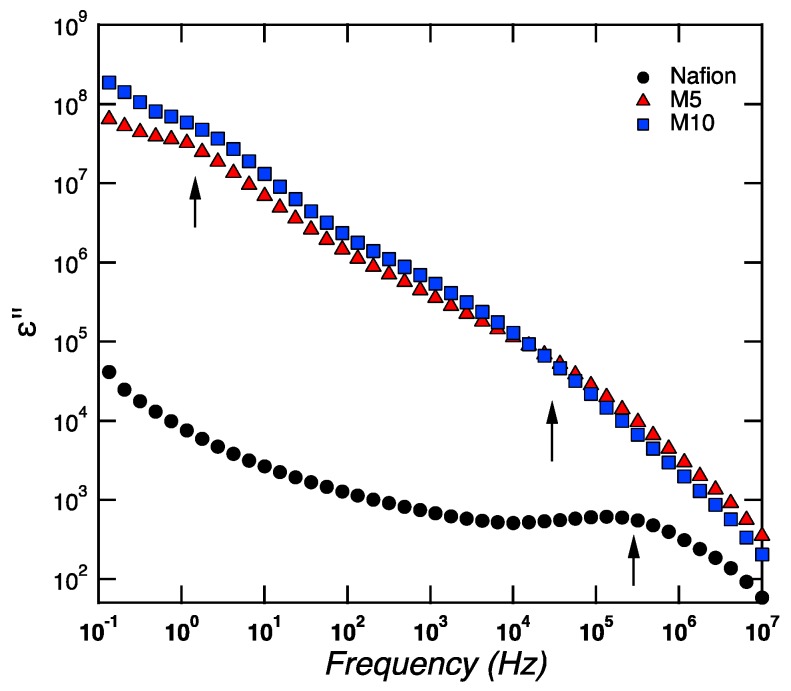
Frequency dependence of the imaginary part of the permittivity for Nafion, M5, and M10, at a given temperature of 20 °C, during cooling.

Figure 5 shows the frequency dependence of the imaginary part of the permittivity (ε′′) as a function of frequency for plain Nafion, M5, and M10, at a given temperature of 20 °C, during cooling.

**Figure 6:**
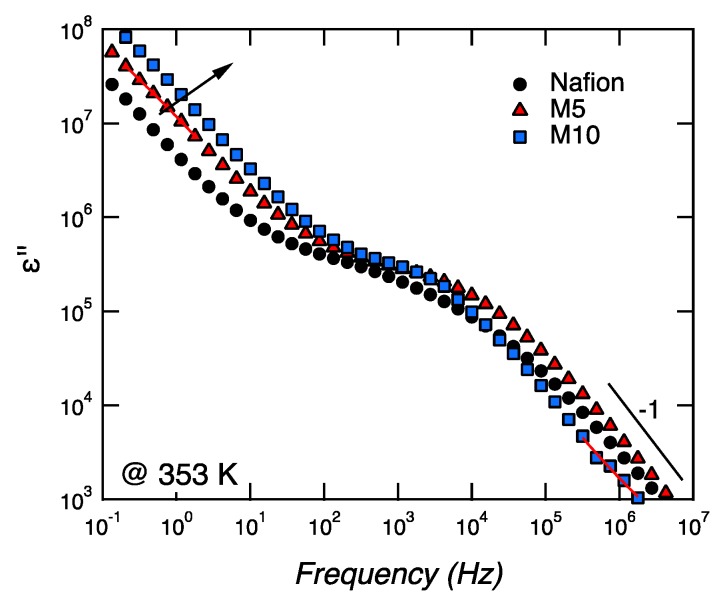
Frequency dependence of the imaginary part of the permittivity for Nafion, M5, and M10, at a given temperature of 80 °C, during heating.

For samplesM5 and M10, two clear dielectric relaxation processes are observed at high and low frequencies. These relaxation processes, called β-relaxations (i.e.,β_1_, β_2_) and generally observed at a low temperature (i.e., lower than *T*_g_), were attributed to conformational changes of the ether group bound to the backbone end of the side-chain or the ether group bound to the sulfonate end of the side-chain [28]. These β-relaxations are completely different from the main structural α-relaxation, which occurs at higher temperatures, around 110 °C, as revealed by DMA measurements, and is associated to the long-range movement of the fluorocarbon domains (i.e., to *T*_g_). In the case of Nafion, one major β-relaxation process is observed at high frequencies, while, at the limit of the lowest frequency investigated, the shape of the curve is reminiscent of the tail of a β-relaxation (peaked at low frequencies outside the frequency window investigated).A similar behavior was found in Nafion membranes investigated by Di Noto et al. [28,29]. Both β-relaxations (i.e.,β_1_, β_2_) become faster with increasing temperature and move to higher frequencies, which implies that, at 80 °C, both are outside the experimental frequency window investigated (see Figure 6). This figure shows that the main structural α-relaxation is not observed within the studied frequency window, but two conductivities are detected instead. These are recognized by the typical slope of −1.

We propose to assign the conductivity observed at low frequencies (i.e., in the range 10^−1^–10^1^ Hz) to localized and slow phenomena, whereas the conductivity at higher frequencies (i.e., in the range 10^4^–10^6^ Hz) is attributed to long-range bulk or dc conductivity. Both these two conductivities are associated to ε’’, with a slope of −1, and become faster with an increasing concentration of CaTiO_3_ (see arrow in Figure 6).

**Figure 7:**
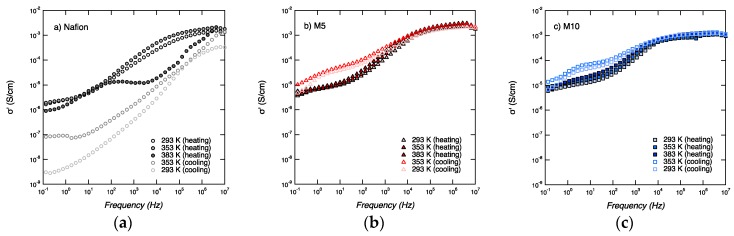
Conductivity (σ’) spectra as a function of frequency for Nafion (**a**), M5 (**b**), and M10 (**c**), during heating and cooling.

The conductivity curves recorded for all three membranes (Nafion, M5, and M10) and over the whole frequency window, as well as for all temperatures (i.e., 20, 80, and 110 °C) are shown in Figure 7, both for the heating and cooling scans.

Two characteristic ranges can be observed, at low (10^−1^–10 Hz) and high (10^4^–10^6^ Hz) frequencies, a behavior similar to that described by Di Noto et al., in the investigation of dry and wet Nafion [28]. 

**Figure 8:**
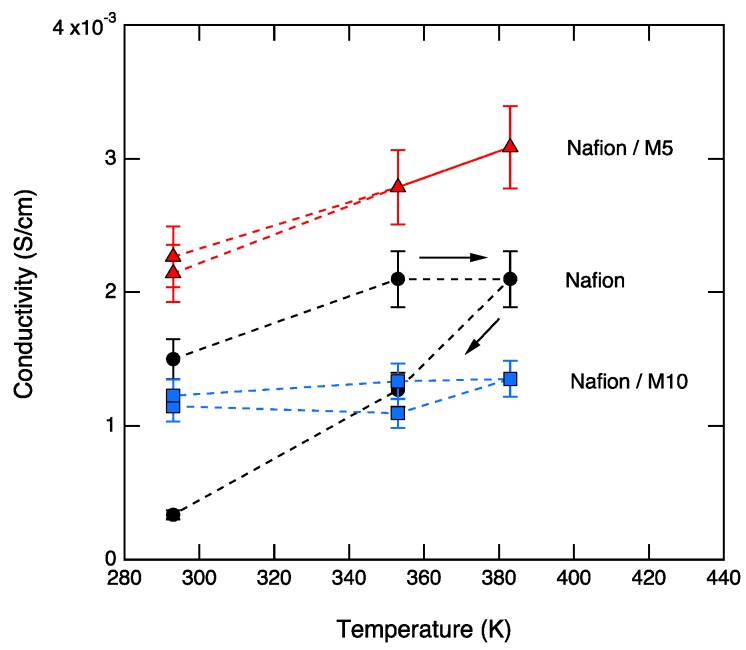
The dc conductivity as a function of temperature for Nafion, M5, and M10, during heating and cooling.

The conductivity at low frequencies (10^−1^–10^1^ Hz) shows a similar general trend for the three samples, increasing with increasing temperatures during heating and decreasing, with decreasing temperature during cooling. However, the conductivity at high frequencies (10^4^–10^6^ Hz) shows different behaviors in the three samples. For Nafion, the conductivity values are lowered by one order of magnitude after heating to 110 °C, which most likely reflects the dehydration of the membrane. For M5, the conductivity values during heating and cooling show a small but detectable change, with the conductivity increasing upon increased temperature. M5 also shows the highest conductivity, reaching a value at 110 °C of 3.1 × 10^−3^ S∙cm^−1^ at 110 °C. For M10, there is no significant change in conductivity during heating and cooling, and a value about 1.2 × 10^−3^ S∙cm^−1^ is measured. These trends are better visualized in Figure 8.

Table 2 shows the dependence of the dc conductivity on composition, for the representative temperature of 110 °C. The three samples all exhibit a reasonably high conductivity (~10^−3^ S·cm^−1^), although M5 displays the highest value, indicating that a too-high concentration of CaTiO_3_ can be detrimental for the conducting property. The conductivity displayed by M5 at 110 °C is higher than that of dry Nafion at the same temperature [28] and slightly lower than, but comparable to, that of Nafion containing sulfated zirconia as the filler (also added at 5 wt.%) [29].

The reported results indicate that composite membranes obtained by adding calcium titanate as filler in a Nafion matrix display interesting properties that ought to be considered for electrochemical applications, such as in PEMFCs. An improved water affinity and enhanced proton conductivity is observed for a low concentration of the filler (around 5 wt.%), whereas higher filler contents (about 10 wt.%) are not beneficial for the thermal and conducting performance of the membrane. This behavior was already reported for other types of filler in composite membranes [24]. In particular, previous results published by Chen et al. [30] show that Nafion membranes containing 5 wt.% of sulphated tin oxide had higher proton conductivity than both the undoped membrane and the membrane with 10 wt.% of filler. Moreover, filler loading around 4–5 wt.% was the most effective, also for Nafion-based composite membranes containing different metal oxide fillers, likely due to the more homogeneous dispersion of the additive within the polymer matrix [8,27]. The issues of inhomogeneous dispersion and non-optimized filler-to-polymer interactions appear to be particularly critical in this work due to the micrometric size of the calcium titanate particles [14], exceeding the dimension of the hydrophilic domains and, possibly, occluding them to some extent. Moreover, a too-high filler concentration could block the ionic channels and impede ionic motion, as also suggested for proton-conducting hybrid membranes containing protic ionic liquids and silica nanoparticles or mesoporous silica nanospheres [31].

## 4. Conclusions

In the present paper, a composite membrane based on a Nafion polymer matrix incorporating a CaTiO_3−δ_ additive is proposed and investigated. Different filler contents, namely two concentrations of 5 and 10 wt.% of the CaTiO_3−δ_ additive, with respect to the dry Nafion content, were considered. The membrane with the lower amount of additive displayed better properties in terms of both water affinity and conductivity. Indeed, our results suggest that a too-high content of additive can be detrimental for these particular properties. However, our results indicate that perovskite oxides can be useful as a water-retention and reinforcing additive in low-humidity proton-exchange membranes.

## Figures and Tables

**Table 1:** ΔH and *T*_onset_ associated to the thermal transition observed by DSC.

Samples	*T*_onset_ (°C)	Δ*H* (J·g^−1^)
N	82	738
M5	81	747
M10	55	380

**Table 2:** Conductivity values (σ) measured for different membranes at 110 °C.

Sample	wt.% CaTiO_3_	σ (mS·cm^−1^)
N	0	2.1
M5	5	3.1
M10	10	1.3
Nafion (wet) [28]	-	10–100
Nafion (dry) [28]	-	0.0001–0.001
S-ZrO_2_/Nafion (dry) [29]	-	1–10
